# Characterization of Chicken *MMP13* Expression and Genetic Effect on Egg Production Traits of Its Promoter Polymorphisms

**DOI:** 10.1534/g3.116.027755

**Published:** 2016-03-09

**Authors:** Zhenjie Yuan, Yuxia Chen, Qiuyue Chen, Miao Guo, Li Kang, Guiyu Zhu, Yunliang Jiang

**Affiliations:** * Department of Genetics, College of Agronomy, Shandong Agricultural University, Taian 271018, China; † Department of Animal Genetics, Breeding and Reproduction, College of Animal Science and Veterinary Medicine, Shandong Agricultural University, Taian 271018, China; ‡ Department of Biology Science and Technology, Taishan University, Taian 271000, China

**Keywords:** chicken, *MMP13*, follicle, polymorphism, age at first laying

## Abstract

Extracelluar matrix undergoes constant remodeling, cell–cell, and cell–matrix interactions during chicken ovarian follicle growth, which is coordinated by matrix metalloproteinases (MMPs), and their associated endogenous inhibitors (TIMPs). Transcriptome analysis revealed upregulation of *MMP13* in sexually mature chicken ovaries. In this study, we found that the expression of *MMP13* in chicken ovary was stably elevated from 60 d to 159 d, and was significantly higher at 159 d than at the other three developmental stages (*P* < 0.05). The expression of *MMP13* mRNA increased from SW (small white follicles) to F5 (fifth largest follicles), then decreased to F1 (first largest follicles), and dramatically increased again in POF1 (newly postovulatory follicles) follicles (*P* < 0.05). The MMP13 protein was localized in stroma cells and primordial follicles of sexually immature chicken ovaries, in the theca cell layers of all sized follicles of sexually mature chicken ovaries. Furthermore, we identified a positive element (positions –1863 to –1036) controlling chicken *MMP13* transcription, and, in this region, six single nucleotide polymorphisms were found and genotyped in chicken populations. In the White Recessive Rock population, hens with *A^–1356^-C^–1079^*/*A^–1356^-C^–1079^* genotype had earlier “age at first laying” than those with *G^–1356^-T^–1079^*/*G^–1356^-T^–1079^* genotype (*P* < 0.05), and exhibited significantly lower transcriptional activity (*P* < 0.01). Collectively, chicken MMP13 plays an important role in ovarian follicle growth and regression, and polymorphisms in its promoter region could be used as molecular markers for improving the trait “age at first laying” in chicken breeding.

The ovary represents a truly dynamic organ system characterized by structuring and restructuring events during the development of the ovarian follicle for ovulation. Follicle development is characterized as continued growth from the initial primordial follicle to a mature follicle that is approximately 400-fold larger. During this process, the extracellular matrix (ECM) requires constant remodeling, and cell–cell and cell–matrix interactions to control the wide range of cellular functions, which is coordinated by matrix metalloproteinases (MMPs) and their associated endogenous inhibitors (TIMPs) (Curry and Osteen 2003; Rodgers *et al.* 2003; Ohnishi *et al.* 2005; Curry and Smith 2006).The exquisite control of the MMP system has been postulated to regulate many of the cyclic changes, such as the follicular development, ovulation, the formation or destruction of vascularized ovarian structure (Fata *et al.* 2000; Curry and Osteen 2001; Ny *et al.* 2002; Smith *et al.* 2002; Page-McCaw *et al.* 2007), and therefore is essential for the proper follicular function in the ovary while simultaneously preserving ovarian integrity.

Unlike mammals, the domestic fowl has only a single left ovary, containing follicles of various sizes and developmental stages, including primordial follicles, primary follicles, prehierarchal follicles, such as small white follicles (SW) and small yellow follicles (SY), hierarchal follicles of F5 to F1, and postovulatory follicles (POFs); the POF in birds fails to form a functional corpus luteum (CL) and rapidly regresses. To maintain this hierarchy and daily ovulation, a follicle is selected from the pool of 6–8 mm SY follicles, and grows into a 40 mm preovulatory follicle in just 5–9 d (Gilbert *et al.* 1983). Follicular maturation also requires yolk deposition, which involves the process of transport, transformation, and deposition of yolk prerequisite material that is conveyed to the oocyte via the vascular system. In addition, the POFs regress shortly after ovulation, and are almost entirely regressed by cellular apoptosis accompanied by proteolysis and dissolution of the ECM within 4–6 d after ovulation (Sudaresan *et al.* 2008). These extensive cyclic changes in the follicular ECM throughout each reproductive cycle are postulated to occur via the action of a cascade of proteolytic events involving matrix metalloproteinases (MMPs) activity (Zhu *et al.* 2014).

MMP13, also known as collagenase-3, initiates the breakdown of the fibrillar collagens that form a key structural element of membranes. In chicken, MMP13 is involved in embryonic membrane remodeling, and corneal development related biochemical and molecular changes (Lei *et al.* 1999; Huh *et al.* 2007). In the angiogenesis system of the chorioallantoic membrane, MMP13 is the only enzyme associated with collagen remodeling (Zijlstra *et al.* 2004). Periodic changes of collagenase-3 have been detected in the rat ovary during the ovulatory process (Balbin *et al.* 1996); however, the role of *MMP13* in chicken follicle growth, ovulation, and ovary function is unclear. In this study, we investigated the expression pattern and cellular localization of MMP13 during the reproductive cycle of hens. We also analyzed the regulatory elements of the chicken *MMP13* promoter, and identified six single nucleotide polymorphisms (SNPs) that are associated with egg production traits in a White Recessive Rock population.

## Materials and Methods

### Birds, sample collection, and sample preparation

Hy-line Brown commercial hens at the ages of 60 d, 90 d, 123 d (sexually immature), and 159 d (sexually mature) were used for analyzing the expression pattern of the chicken *MMP13* gene. All birds had free access to water and feed. The hens were housed in separate cages under a daily light period of 14 hr. Hens were killed by cervical dislocation, then ovary and follicles at different stages of development were collected and stored in liquid nitrogen immediately. The whole ovary was harvested for RNA or protein extraction for comparing ovarian MMP13 expression between hens of different developmental stages. Follicles with varied sizes, including SW, F5, F3, F1, and POF1 follicles, were manually dissected out for RNA or protein extractions for comparing chicken MMP13 expression between different follicles.

Chicken populations of White Recessive Rock (*n* = 510), Hy-line brown (*n* = 45), Wenchang (*n* = 53), Wenshang Barred (*n* = 46), and Jining Bairi (*n* = 37), which were randomly sampled from their respective breeding populations, were used for polymorphism analysis. The White Recessive Rock population (*n* = 510) was also used for association analysis of diplotype and egg production traits. Egg production traits, including age at first laying, and egg number at 32 wk, were recorded individually. Sampling was carried out by collecting blood from the wing vein, and storing it at –20° until delivered to the laboratory. Genomic DNA was extracted from the blood sample using a DNA Extraction mini kit (Tiangen, Beijing, China), and stored in TE (pH 8.0) at –20°. The birds were handled and treated according to the Animal Care and Use Committee of Shandong Agricultural University.

### Total RNA extraction and real-time quantitative PCR

Total RNA was extracted from all tissues using an RNA extraction kit DP419 (Tiangen). The amount and integrity of isolated total RNA were measured using a spectrophotometer (Eppendorf, Hamburg, Germany), and checked by loading total RNA onto a 1% agarose gel that was stained with ethidium bromide. The cDNA was synthesized using a PrimeScript RT reagent kit with a gDNA Eraser (TaKaRa, Dalian, China) from 1 μg of total RNA mixed with 1 μl of oligo (dT)18 primer, 1 μl of PrimerScript RT Enzyme Mix I, 4 μl of 5× Prime Script Buffer, and RNase-free ddH_2_O up to 20 μl, and incubated at 37° for 15 min and 85° for 5 sec. The resultant cDNA was stored at –20° for mRNA expression analysis. Real-time quantitative RT-PCR (qRT-PCR) was conducted on an MX3000p (Stratagene, La Jolla, CA) in a 15-μl volume containing 7.5 μl of 2× SYBR Premix Ex *Taq* II (TaKaRa, Dalian, China), 0.3 μl of 50× Rox Reference Dy II, 0.2 μl of each forward and reverse primers (10 μM, β-actin-F/R, MMP13-F/R, and VEGFA-F/R in Table 1), and 1.5 μl of cDNA at a dilution of 1:8 according to the following program: 95° for 30 sec to activate the reaction, and 95° for 5 sec, 56° for 20 sec, and 72° for 15 sec for 40 cycles. Melting curves were used to confirm the specificity of each product, and the efficiency of the PCR was determined by analysis of twofold serial dilutions of cDNA. The PCR efficiency was close to 100%, allowing the use of the 2^–ΔΔCt^ method for the calculation of relative gene expression (Livak and Schmittgen 2001). All qRT-PCRs were carried out with negative controls.

**Table 1:** Primers used for real-time quantitative RT-PCR and polymorphism analyses and plasmid construction of chicken *MMP13* gene

Primer Name	Sequence (5′–3′)	Annealing Temperature (°C)	Size (bp)
MMP13-F	TTTGGATTAGAGGTGACGG	56	253
MMP13-R	CCACTTCGTATTCTGGTGA		
β-actin-F	TGGATGATGATATTGCTGC	56	251
β-actin-R	ATCTTCTCCATATCATCCC		
VEGFA-F	GGAAGCCCAACGAAGTTA	55	231
VEGFA-R	CGCTATGTGCTGACTCTGAT		
snpMMP13-F	ACAATCCCAGTTCCCTCAG	58	807
snpMMP13-R	GCCCAGGCTTGTATCACT		
pGL3-MMP13-1F	CGACGCGTGTGCTGTGCTGTAAGTTGTCTT		
pGL3-MMP13-2F	CGACGCGT CACTTCTCCCATTCTTCCAT		
pGL3-MMP13-3F	CGACGCGT ACAATCCCAGTTCCCTCAG		
pGL3-MMP13-4F	CGACGCGT AGTGATACAAGCCTGGGC		
pGL3-MMP13-5F	CGACGCGT GTCATGTCCTATTATGTACTCC		
pGL3-MMP13-R	GGAGATCTTTTGTGAAGTCTGAAGCCC		

All forward primers contained an *Mlu*I site at their 5′-ends, and the reverse primers had an added *Bgl*II site at their 5′-ends (underlined).

### Western blotting

Total protein was extracted from homogenized ovaries and different developing follicles using the Cell Lysis Reagent (Fermentas). Protein concentration was determined by the bicinchoninic acid assay (BCA Protein Array kit, Tiangen). The mouse anti-rat MMP13 monoclonal antibody was produced by Chemicon (Temecula, CA). An equal amount of protein was separated by 10% SDS gel electrophoresis under denaturing and nonreducing conditions, after which the proteins were transferred to a polyvinylidene fluoride membrane, and then incubated (1 hr, 37°) with the MMP13 (1:250) antibodies in a 5% bovine serum albumin/PBS solution. After washing in PBST (1000 ml PBS:500 μl Tween-20), the blots were incubated with horseradish peroxidase-conjugated goat anti-mouse immunoglobulin G antibody (1:1000; Abcam) in a 5% bovine serum albumin/PBS solution (1 hr, 37°). Specific binding was visualized using diaminobenzidine (Tiangen).

### Immunohistochemistry

Ovaries and follicles were collected from 45-d-old (sexually immature) and 159-d-old (sexually mature) hens. Tissues were fixed in 10% buffered formalin and paraffin-embedded, and then cut into 5 μm tissue sections. All the sections were deparaffinized, rehydrated through a graded ethanol series, boiled in 10 mM sodium citrate buffer, quenched in 3% hydrogen peroxide, and blocked with 10% goat serum for 20 min. Next, the slides were incubated with mouse anti-rat MMP13 monoclonal antibody (1:50) for 2 hr at 37°. Then, the sections were incubated with the biotinylated secondary antibody and avidin-biotin-peroxidase complex for 0.5 hr according to the Histostain-plus kit instructions (Zhongshan Golden Bridge Biotechnology, China). Finally, immunoprecipitates were visualized by incubation with a diaminobenzidine kit (Zhongshan Golden Bridge Biotechnology). The sections were counterstained with hematoxylin after immunostaining, dehydrated, and covered. Negative control staining was performed using PBS instead of primary antibody. No specific staining was found on the control slides.

### Plasmid construction

The 5′-regulatory region of chicken *MMP13* gene was inserted upstream of the firefly luciferase gene of the pGL3-Basic vector (Promega, Madison, WI) to generate reporter plasmids. To construct serial deletion promoter reporters, five forward primers (from pGL3-MMP13-1F to pGL3-MMP13-5F, Table 1), and one reverse primer (pGL3-MMP13-R, Table 1) located downstream of the transcription start site of chicken *MMP13* gene were synthesized. All forward primers contained an *Mlu*I site at their 5′-ends, and the reverse primers had an added *Bgl*II site at their 5′-ends. The PCR amplification of the chicken *MMP13* promoter was carried out with high-fidelity *Taq* DNA polymerase PrimeStar (TaKaRa, Dalian, China) using the chicken genome as template, and all PCR fragments were inserted into pGL3-Basic between the *Mlu*I and *Bgl*II restriction sites to generate 5′ serially deleted promoter constructs. The PCR products and pGL3-Basic vector were separately digested with the same restriction enzyme for 0.5 hr at 37°, and then the two purified products were ligated for 30 min at 22°. To evaluate the effect of the SNPs (g.–1356 G > A, g.–1128 A > G, g.–1094 C > A, g.–1079 T > C) on *MMP13* promoter activity, two reporter plasmids (wt-MMP13 and mut-MMP13 constructs), which encompassed the core promoter region, were constructed with individuals of *G*^-1356^*A*^-1128^*C*^-1094^*T*^-1079^/*G*^-1356^*A*^-1128^*C*^-1094^*T*^-1079^ genotype and *A*^-1356^*G*^-1128^*A*^-1094^*C*^-1079^/*A*^-1356^G^-1128^*A*^-1094^*C*^-1079^ genotype as a template, respectively. PCR amplification was performed in 20 μl volumes containing 4 μl of 5× prime STAR HS Buffer, 1.6 μl (2.5 mM) of dNTPs (TaKaRa, Dalian, China), 0.2 μl of prime STAR HS polymerase (5 U/μl) (TaKaRa, Dalian, China), 0.5 μl of each forward (pGL3-MMP13-3F) and reverse (pGL3-MMP13-R, Table 1) primers (10 μM), 1 µl genomic DNA(50–100 ng), and 12.7 μl of nuclease ddH_2_O, and run on a Mastercycler gradient (Eppendorf, Germany) according to the following program: 98° for 10 min, 35 cycles of 98° for 10 sec, annealing at 58° for 30 sec, and 72° for 1 kb/min, and final extension at 72° for 5 min. All constructs were sequenced in both directions to confirm the authenticity of the sequences. Plasmids were reproduced in *Escherichia coli* DH5α, and purified using the Endo-Free Plasmid Purification Kit (Tiangen).

### Cell culture and luciferase assay

The F1, F2, and F3 follicles of the ovaries of egg-laying hens were separated from the ovaries and placed in PBS (HyClone). Theca cells were isolated according to the protocol described in Gilbert *et al.* (1977). The theca cells were dispersed by treatment with 0.1% collagenase II at 37° for 25 min with gentle agitation in a flask. After centrifugation, the cells were suspended in culture medium (M199 with 10% fetal bovine serum, and 1% penicillin/streptomycin), and subsequently seeded in 24-well culture plates at a density of 1 × 10^6^/well. The number of viable cells (90%) was estimated using Trypan blue. Cells were cultured at 38.5° in a water-saturated atmosphere of 95% air and 5% CO_2_ for 24 hr.

Theca cells grown to 80% confluency were transfected with plasmids using NanoFectin Transfection Reagent (Excell Biology, Shanghai, China) following the supplier’s protocol. Different promoter–reporter fusion plasmids (800 ng), and 25 ng of the pGL4.74 control vector (Promega, Madison, WI) were cotransfected into theca cells; 12 hr later, the cells were maintained in M199 medium without fetal bovine serum. The cells were then washed twice in phosphate-buffered saline and lysed in 1× Passive Lysis Buffer. Luciferase activity was measured in supernatant extracts with the Dual-Luciferase Reporter Assay System (Promega, Madison, WI). Cotransfection with pGL4.74 plasmid DNA was carried out to normalize transfection efficiencies. The transfections were performed at least in triplicate for each construct.

### Polymorphism analysis

PCR amplification was performed in 20 µl volumes containing 2 µl of 10× Ex-buffer, 1.6 μl (2.5 mM) of dNTPs (TaKaRa, Dalian, China), 0.1 μl (5 U/μl) of Ex-Taq DNA polymerase (TaKaRa, Dalian, China), 0.4 μl of each forward and reverse primers (10 μM, snpMMP13-F/R in Table 1) designed according to the chicken *MMP13* gene (GenBank accession No. NC_006088.3), 1 µl genomic DNA pools (50–100 ng), and 14.5 μl of nuclease ddH_2_O, and run on a Mastercycler gradient (Eppendorf, Germany) according to the following program: 94° for 4 min, 35 cycles of 94° for 30 sec, annealing at 58° for 30 sec, and 72° for 45 sec, and final extension at 72° for 5 min. Fragments of 807 bp were resolved by electrophoresis on a 1% agarose gel, purified with AxyPrep DNA Gel Extraction Kit (Axygen, Union City, CA), and sequenced using forward primer. Sequences were aligned with the reference sequence (GenBank accession No. NC_006088.3) to identify nucleotide changes using the DNAMAN program.

### Statistical analysis

The SHEsis software (http://analysis.bio-x.cn) was used to analyze the pairwise linkage disequilibrium and haplotype frequency. The genotype and allelic frequencies, Hardy-Weinberg equilibrium χ^2^ test, polymorphism information contents (PIC), heterozygosities (*H*_e_), and the effective population of the allele (*N*_e_), were calculated using Tools for Population Genetic Analyses software (http://www.marksgeneticsoftware.net/tfpga.htm). Haplotypes were constructed in chicken populations using the PHASE v2.0 program. The associations of *MMP13* genotypes with egg production traits including age at first laying, and egg number at 32 wk, were analyzed in White Recessive Rock hen populations that were derived from the same chicken farm using the General Linear Model of SAS (version 9.2; Cary, NC). The linear model is represented as follows: *Y*_ij_ = μ + *G*_i_ + *e*_ij_, where *Y*_ij_ is the phenotypic value of traits, μ is the population mean, *G*_i_ is the fixed effect of genotype, and *e*_ij_ is the random error effect. For qRT-PCR analysis, western blotting analysis, and luciferase assay, differences between the experimental groups were evaluated by ANOVA, followed by Duncan’s multiple range test (*P* < 0.05) using the General Linear Model procedure of SAS (SAS version 9.2; Cary, NC). All the expressions were repeated at least three times, and all data were presented as the mean ± SEM.

### Data availability

Supplementary file File S1 contains genotype and phenotype data of chicken individuals used for diplotype and egg production trait association analysis. Sequence data are available at GenBank and the accession number is 1971458461 for SNP g. -1356G>A.

## Results

### Expression of MMP13 in chicken ovaries of different developmental stages

**Figure 1:**
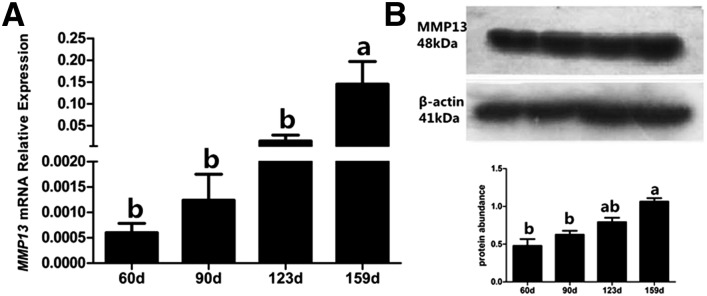
Expression of MMP13 in ovaries from 60-, 90-, 123-, and 159-d-old hens. (A) Messenger RNA expression of *MMP13* was analyzed by real-time PCR. (B) Expression of MMP13 protein was analyzed by Western blot analysis. A band of approximately 48 kDa corresponding to the molecular mass of MMP13 was detected. β-actin (41 kDa) was used as the loading control. Data are presented as mean ± SEM from at least four independent experiments. Bars with different superscript letters are significantly different (*P* < 0.05).

Illumina/solexa sequencing revealed that the expression of chicken *MMP13* was significantly higher in the ovaries of laying hens than in prepubertal hens (Zhu *et al.* 2014). This expression dynamic was also validated in Jining Bairi and Hy-line hens (Supplemental Material, Figure S1). In this study, we further analyzed the expression of *MMP13* mRNA in the ovary of Hyline-brown hens during development. A stably elevated mRNA expression of chicken *MMP13* from 60 d to 159 d was observed, and, at 159 d, it was significantly higher than at the other three developmental stages (*P* < 0.05) (Figure 1A). Similarly, the protein level of chicken MMP13 in the ovary was also increased during chicken development, reaching the highest level at 159 d (*P* < 0.05) (Figure 1B).

### Expression of MMP13 mRNA and protein during chicken follicular development

**Figure 2:**
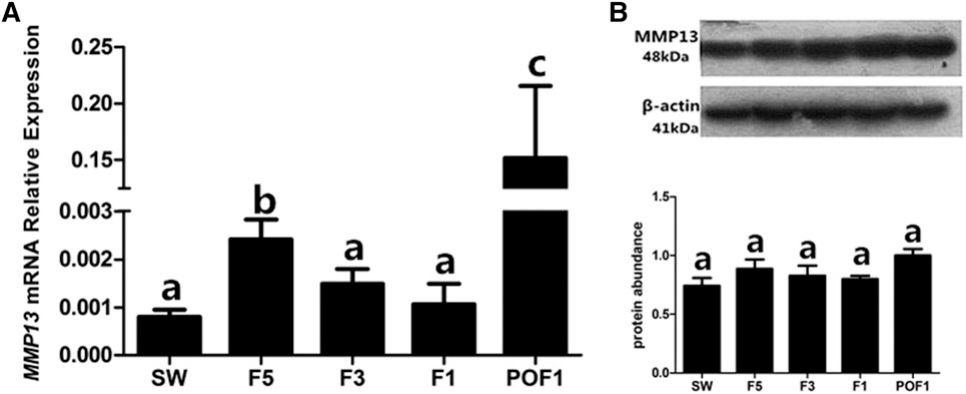
Expression of MMP13 in different follicles from 159-d-old hen ovaries. (A) Expression of *MMP13* mRNA in small white follicles (SW), fifth largest follicles (F5), third largest follicles (F3), first largest follicles (F1), and newly postovulatory follicles (POF1). (B) Expression of chicken MMP13 protein was analyzed by Western blot analysis. β-actin (41 kDa) was used as the loading control. Data are presented as mean ± SEM from at least four independent experiments. Bars with different superscript letters are significantly different (*P* < 0.05).

The expression of *MMP13* mRNA increased from SW to F5, then decreased to F1 and dramatically increased again in POF1 follicles (*P* < 0.05) (Figure 2A). The expression of MMP13 protein among SW, F5, F3, F1 to POF1 follicles was not significant (*P* > 0.05) (Figure 2B).

### Spatiotemporal changes in the localization of chicken MMP13 protein

**Figure 3:**
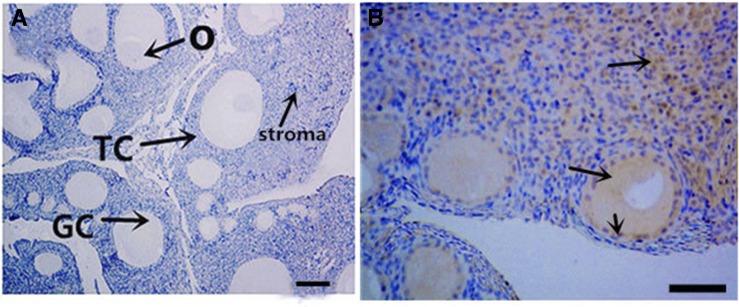
Immunohistochemistry of chicken MMP13 expression in 45-d-old hen ovaries. (A) Negative control with PBS used in place of the primary antibody. (B) Localization of MMP13 protein in the ovaries of 45-d-old chickens; arrowheads indicate the position of strongly stained protein. GC, granulosa cells; TC, theca cells; O, oocyte. Bar for (A): 100 μm; (B): 200 μm.

**Figure 4:**
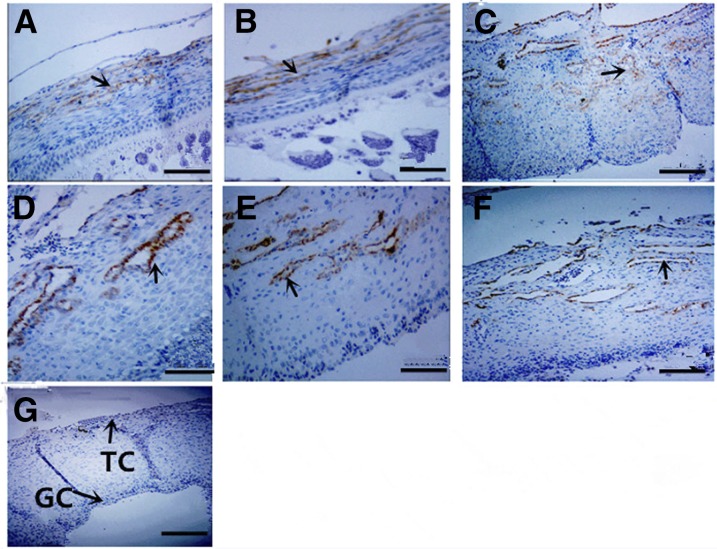
Immunohistochemistry detection of chicken MMP13 protein in different follicles from the 159-d-old hen ovaries. (A) SW, (B) SY, (C) F5, (D) F3, (E) F1, (F) POF1, and (G) negative control with PBS used in place of the primary antibody. Arrowheads indicate the position of strongly stained protein. GC, granulosa cells; TC, theca cells. Bar for (A–G): 200 μm

**Figure 5:**
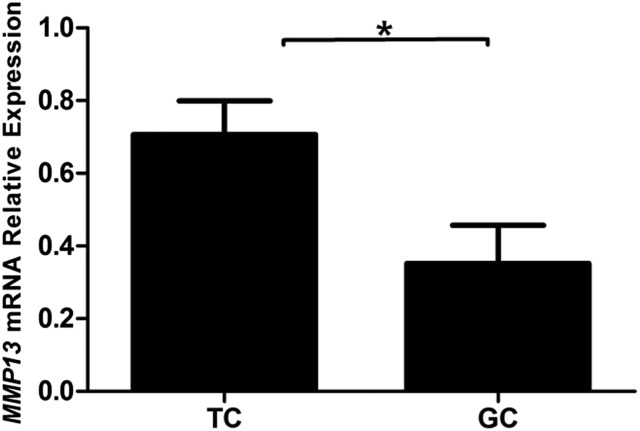
mRNA expression of chicken *MMP13* in theca cells (TC) and granulosa cells (GC). * *P* < 0.05.

Cellular localization of MMP13 protein was examined in chicken sexually immature (45-d-old) and mature (159-d-old) ovaries. In sexually immature chicken ovaries, MMP13 protein staining was observed in stroma cells and primordial follicles (Figure 3). In sexually mature chicken ovaries, MMP13 protein is localized mainly in theca cell layers of all sized follicles, and in follicles of F5, F3, F1, and POF1, its expression is restricted mainly to blood vessels of theca cell layers (Figure 4). In chicken preovulatory follicles, *MMP13* mRNA level was significantly higher in theca cells than in granulosa cells (Figure 5).

### Polymorphisms in the critical promoter region of the chicken MMP13 gene

**Figure 6:**
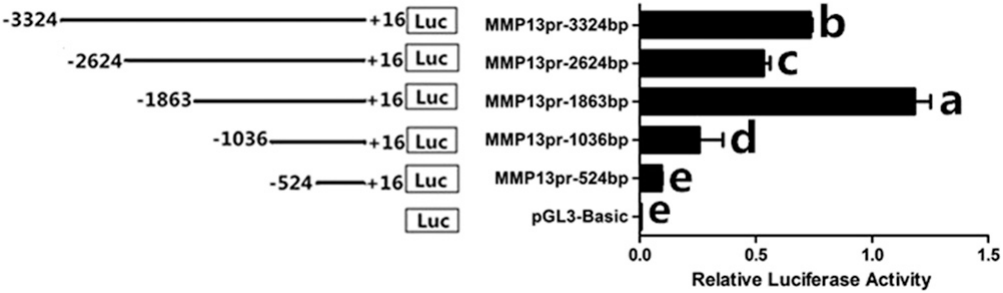
Luciferase assay of chicken *MMP13* gene promoter transfections. Schematic structure of various progressive deletion in the 5′ flanking region of the chicken *MMP13* gene, and the activity of the corresponding truncated chicken *MMP13* promoters. Luciferase vectors with progressive 5′ deletions were constructed and transiently transfected into theca cells as described. The pGL3-basic vector was used as a control. A renilla luciferase reporter plasmid was used as the internal control to correct for transfection efficiency. Data are presented as mean ± SEM from at least four replicates for each construct. Means with the different lowercase letters within the same column are significantly different (*P* < 0.05).

**Figure 7:**
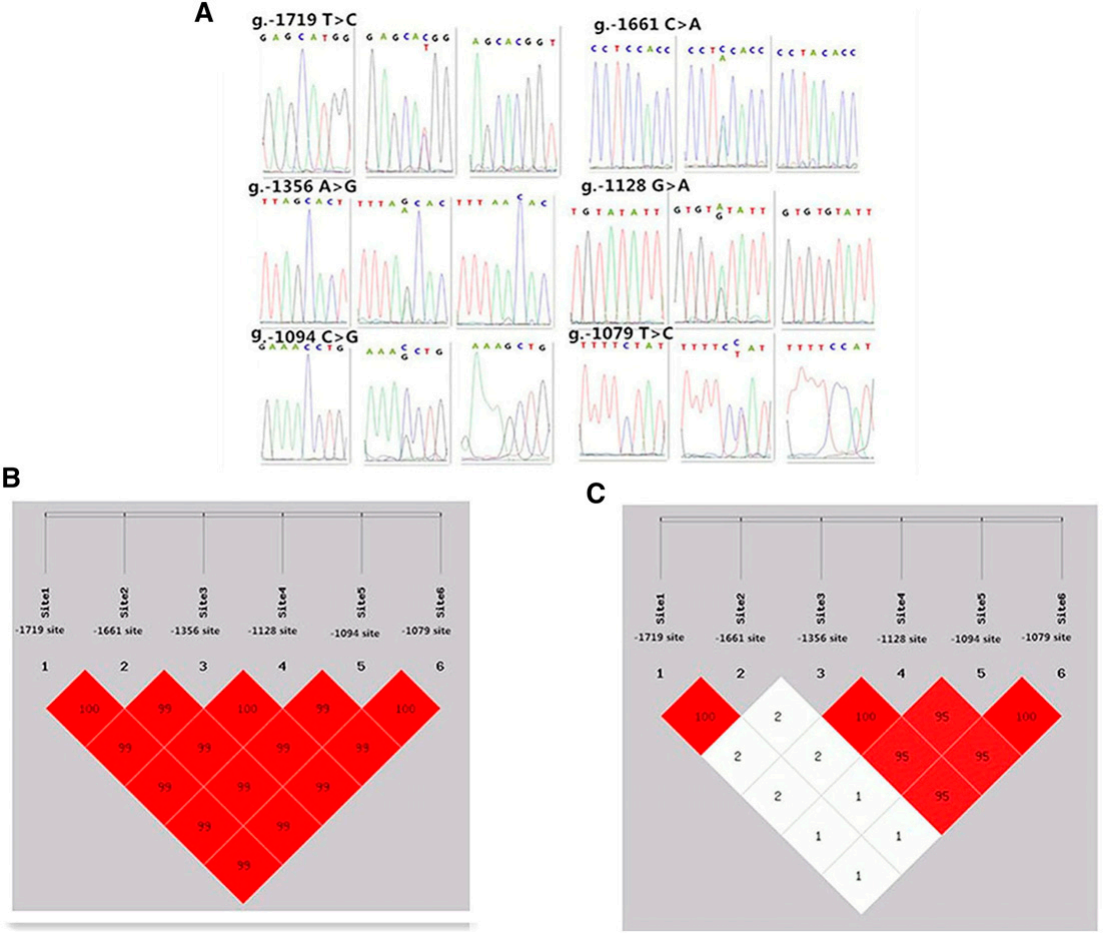
Six single nucleotide polymorphisms identified in the promoter region from −1863 bp to −1036 bp of chicken *MMP13* gene (A) and the linkage disequilibrium (LD) result by the online software SHEsis analysis (B and C). Normalized LD was shown as *D*’ (B) and *R*^2^ (C), the values of *D*’ and *R*^2^ are in the range between 0 and 100%. *D*’ can be seen as a frequency independent metric, and *R*^2^ is a measure of frequency. *D*’ and *R*^2^ value are 100% when the chain is in complete LD.

We further identified a critical region controlling the transcription of chicken *MMP13* gene, and found that deletion from −1863 bp to −1036 bp significantly decreased the promoter activity as assayed by luciferase activity (*P* < 0.05) (Figure 6). In this region, six single nucleotide polymorphisms (SNPs), *i.e.*, g. –1719 T > C (rs731299043), g.–1661 C > A (rs314003171), g.–1356 G > A(ss1971458461) identified by this study, g.–1128 A > G (rs316193109), g.–1094 C > G (rs315077856), and g.–1079 T > C (rs312778897) were found, among which complete linkage disequilibrium exist between g.–1719 and g.–1661, g.–1356 and g.–1128, and g.–1094 and g.–1079 loci, respectively (Figure 7). At SNPs g.–1719 T > C, g.–1661 C > A, g.–1356 G > A, g.–1128 A > G, g.–1094 C > G, g.–1079 T > C, alleles *T*, *C*, *G*, *A*, *C*, and *T* were predominant in White Recessive Rock, Hy-line brown, Wenchang, Jining Bairi, and Wenshang Barred chicken populations, respectively (Table 2).

**Table 2:** Allele frequency of six SNPs in the promoter region of the chicken *MMP13* gene

Locus or Haplotype	Allele	White Recessive Rock (*n* = 510)	Hy-Line Brown (*n* = 45)	Wenchang (*n* = 53)	Jining Bairi (*n* = 37)	Wenshang Barred (*n* = 46)
g.–1719T > C	T	0.917	1.000	0.774	0.838	0.935
g.–1661C > A	C	0.917	1.000	0.774	0.838	0.772
g.–1356G > A	G	0.819	0.600	0.953	0.622	0.989
g.–1128A > G	A	0.819	0.600	0.679	0.595	0.804
g.–1094C > G	C	0.825	0.600	0.755	0.676	0.989
g.–1079T > C	T	0.825	0.600	0.755	0.676	0.837

### Associations of diplotypes of chicken MMP13 gene with egg production traits

To analyze the associations of SNPs in the chicken *MMP13* promoter region with egg production traits, haplotypes were constructed using the two SNPs of g.–1356 G > A and g.–1079 T > C. Three haplotypes of A (*G*^-1356^/*T*^-1079^), C (*A*^-1356^/*T*^-1079^), and D (*A*^-1356^/*C*^-1079^) were detected in the White Recessive Rock population. Due to the fact that the frequency of haplotype C was less than 0.01, association with egg production traits was analyzed only with diplotypes AA, AD, and DD. The results indicated that hens with diplotype DD had earlier age at first laying than those with diplotype AA and AD (*P* < 0.05), but its effect on egg number at 32 wk was not significant (*P* > 0.05) (Table 3).

**Table 3:** Effect of the diplotypes of the SNPs g.–1356 and g.–1079 on laying traits in the White Recessive Rock chicken population

Diplotype	Number	Age at First Laying	Egg Number at 32 wk
AA	335	176.27 ± 0.48 a	31.65 ± 0.39
AD	162	176.73 ± 0.69 a	31.35 ± 0.55
DD	8	168.86 ± 3.33 b	35.57 ± 2.62

Means with the different lowercase letters within the same column are significantly different (*P* < 0.05).

### Genetic effect of the SNPs on chicken MMP13 expression

**Figure 8:**
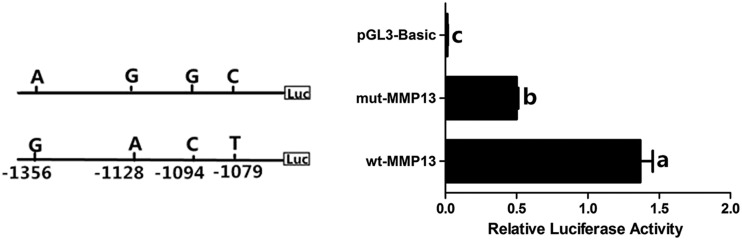
Effect of the six single nucleotide polymorphisms on chicken *MMP13* transcriptional activity in theca cells. Data are presented as mean ± SEM from at least four replicates for each construct.

To further investigate the genetic effect of promoter SNPs on chicken *MMP13* expression, the transcriptional activity of the 5′-flanking region of the *MMP13* gene, which contains the *G*^-1356^/*A*^-1128^/*C*^-1094^/*T*^-1079^ and *A*^-1356^/*G*^-1128^/*G*^-1094^/*C*^-1079^ (designated as wt-MMP13 and mut-MMP13), respectively, was compared. As shown in Figure 8, the wt-MMP13 promoter showed significantly higher transcriptional activity than mut-MMP13 (*P* < 0.05).

## Discussion

In chicken, during follicle growth and ovulation, MMPs and their associated endogenous inhibitors, TIMPs, play critical roles in ECM remodeling. Our previous study has characterized the expression and regulation mechanism of chicken *MMP1*, *MMP3*, and *MMP9* in the ovary and ovarian follicles (Zhu *et al.* 2014), and identified an indel polymorphism in the promoter region of the chicken *MMP9* that is associated with egg number at 28 wk (Zhu and Jiang 2014). From a transcriptome study, we also found that the *MMP13* mRNA is elevated in sexually mature chicken ovary (Zhu *et al.* 2014); therefore, in this study, we further analyzed the expression dynamics and regulation of chicken *MMP13* transcription, and identified a haplotype that is associated with the trait “age at first laying”.

MMP13, also called collagenase-3, was first cloned from a cDNA library derived from a breast tumor (Freije *et al.* 1994). In this study, we first validated the expression dynamics of *MMP13* mRNA in Jining Bairi and Hy-line hens by qRT-PCR (Figure S1), and found that, in both breeds, its mRNA expression level was significantly increased in sexually matured ovaries (140 d for Jining Bairi hens, 300 d for Hy-line hens) compared with sexually immature ovaries (90 d for Jining Bairi hens, 70 d for Hy-line hens), which is consistent with transcriptome results (Zhu *et al.* 2014). Second, we analyzed the expression dynamics of chicken MMP13 during chicken development from 60-d-old to 159-d-old hens, and found that *MMP13* expression in the ovary of 159-d-old hens was significantly higher than other developmental stages, suggesting that MMP13 plays a role in the proper function of the ovary in laying hens. When expression of MMP13 was further analyzed in different sized follicles of sexually mature Hy-line hens, two expression peaks in F5 and POF1 follicles at mRNA level, and one in POF1 follicles at the protein level were revealed, suggesting MMP13 is an important enzyme in follicle selection and follicle regression. In rat ovary, the expression of collagenase-3 mRNA was upregulated by 32-fold at 48 h after eCG injection; MMP13 is likely responsible for the extracellular matrix remodeling associated with follicular growth (Cooke *et al.* 1999), and, during formation and regression of the rat CL, collagenase-3 had a separate expression pattern, being expressed only in the regressing CL, suggesting a specific role of MMP13 in luteal regression (Liu *et al.* 1999). In chicken, although formation of a CL is not observed, follicle regression is a similar process; consistently, a higher expression of MMP13 was also observed in POF1. This suggests that chicken MMP13 is also important in follicle regression.

The distribution of chicken MMP13 protein in the stroma cells of sexually immature chicken ovary, and in the theca cells of sexually mature chicken ovary, suggest that MMP13 is involved in the action of a cascade of proteolytic events for folliculogenesis, and proper follicle function. In ovarian follicles, the expression of *MMP13* is significantly higher in theca cells than in granulosa cells. These results suggest that the MMP13 is secreted mainly by theca cells to regulate follicle growth and ovulations. In accordance with the results of this study, MMP13 was also shown to be localized in rat ovary (Balbin *et al.* 1996), and in the granulosal and thecal layers of bovine preovulatory follicles (Bakke *et al.* 2004). In addition, the expression of *MMP13* is stimulated by LH in rat (Komar *et al.* 2001), PGF2α in sheep (Ricke *et al.* 2002), and GnRH in cattle (Bakke *et al.* 2004). In broiler hens consuming feed *ad libitum* compared to feed-restricted hens, granulosa cells from F1 follicles have less collagenase-3-like gelatinolytic activity (Liu *et al.* 2014), consistent with the fact that less ovulation of mature follicle occurs in the former, emphasizing the importance of MMP13 in chicken follicle maturation. Whether the expression of MMP13 in laying hens is regulated by the aforementioned hormones requires further investigation. Studies have indicated that MMP13 plays a role in the processes of vascularization and ossification (Inada *et al.* 2004; Negev *et al.* 2008), and induction and expression of MMP13 coincided with the onset of angiogenesis and blood vessel formation in the chorioallantoic membrane (Zijlstra *et al.* 2004). We also found that chicken *MMP13* was expressed in the veins of the theca cell layer; consistently, the expression of *VEGFA* mRNA exhibits a similar pattern (Figure S2), which suggests that MMP13 is likely involved in angiogenesis. Further study is required to clarify the relationship between MMP13 and VEGFA in chicken follicle growth.

As the expression dynamics of chicken *MMP13* accompany follicle growth and ovulation, we set out to analyze the regulatory mechanism of *MMP13* transcription and identified a critical region stimulating its transcription. In this region, six SNPs were identified, among which haplotype D caused by SNPs at –1356 and –1079 sites has a positive effect on the age at first laying trait in the White Recessive Rock population, and hens of diplotype DD are expected to lay the first egg about 8 d earlier (Table 3). Moreover, luciferase assay indicated that the promoter of a chicken *MMP13* gene harboring *G*^-1356^/*A*^-1128^/*C*^-1094^/*T*^-1079^ has a higher transcriptional activity than one harboring *A*^-1356^/*G*^-1128^/*G*^-1094^/*C*^-1079^. Using Genomatix Online Software (http://www.genomatix.de/index.html), transcription factors that can bind to these *cis*-elements were predicted. The two polymorphic alleles may have different binding affinity for MEL1 DNA-binding domains 2 (MELS), Kruppel-like factor 7 (KLF7), and Grainyhead-like2 (Grhl2) at g.−1356 (G > A), g.−1128 (A > G), and g.−1094(C > G), respectively. MEL1 encodes a zinc finger protein, and overexpression of a MEL1S lacking PR domain blocked granulocytic differentiation, acting as one of the causative factors in the pathogenesis of myeloid leukemia (Nishikata *et al.* 2003). Chicken KLF7 contains three C_2_H_2_-type zinc fingers domain at the C-terminus (Zhang *et al.* 2013) that are important regulators of cell proliferation and differentiation in several different organ systems (Laub *et al.* 2005; Lei *et al.* 2005; Caiazzo *et al.* 2010). Grhl2 belongs to the grainyhead-like transcription factor family, plays an important role in growth and development, neural tube closure, and epithelial cell differentiation (Werth *et al.* 2010; Pyrgaki *et al.* 2011; Senga *et al.* 2012). Whether these SNPs affect transcription of chicken *MMP13* by interfering with the binding of MELS, KLF7, and Grhl2 remains to be determined.

In conclusion, in laying hens, the expression of both chicken *MMP13* mRNA and protein was increased significantly in the ovary of 159-d-old hens and POF1 follicles, and chicken MMP13 protein was predominantly expressed in theca cells of sexually mature ovaries. Positive *cis*-acting element controlling chicken *MMP13* transcription was identified, and, in this region, six SNPs were found and genotyped in chicken populations. In the White Recessive Rock population, hens with *A*^–1356^*-C*^–1079^ haplotype had earlier age at first laying than those with the *G*^–1356^*-T*^–1079^ haplotype, and exhibited lower transcriptional activity by luciferase assay. These results collectively suggest that MMP13 plays an important role in chicken follicle growth and POF regression, and that polymorphisms in its promoter region could be used as molecular markers for improving the trait age at first laying in chicken breeding.

## Supplementary Material

Supplemental Material
