# Supplementary material for: Characterization of Chicken MMP13 Expression and Genetic Effect on Egg Production Traits of Its Promoter Polymorphisms
Source: G3 (Bethesda). 2016 Mar 9;6(5):1305–12. doi: 10.1534/g3.116.027755 (PMC4856082; doi:10.1534/g3.116.027755)
Supplement: Supplemental Material [file supp_6_5_1305__index.html]

Characterization of Chicken MMP13 Expression and Genetic Effect on Egg Production Traits of Its Promoter Polymorphisms — Supplemental Material 

# Characterization of Chicken *MMP13* Expression and Genetic Effect on Egg Production Traits of Its Promoter Polymorphisms

## Supplemental Material for Yuan *et al.*, 2016

**Files in this Data Supplement:**

- Figure S1 - Expression of *MMP13* mRNA in the ovary of (A) Hy-line hens and (B) Jining Bairi hens. Data are presented as mean ± SEM from at least four independent experiments.\*\*P <*0.01*. (.jpg, 197 KB)
- Figure S2 - Expression of *VEGFA* mRNA in the small white follicles (SW), the small yellow follicles (SY), the fifth largest follicles (F5), the third largest follicles (F3), the first largest follicles (F1) and the newly post-ovulatory follicles (POF1) of 159-day-old hens. Data are presented as mean ± SEM from at least four independent experiments. Bars with different superscript letters are significantly different (P <*0.05*). (.jpg, 149 KB)
- File S1 - This file contains genotype and phenotype data of chicken individuals used for diplotype and egg production trait association analysis. (.xlsx, 38 KB)
