# Supplementary figures and images for: Characterization of Chicken MMP13 Expression and Genetic Effect on Egg Production Traits of Its Promoter Polymorphisms
Source: G3 (Bethesda). 2016 Mar 9;6(5):1305–12. doi: 10.1534/g3.116.027755 (PMC4856082; doi:10.1534/g3.116.027755)

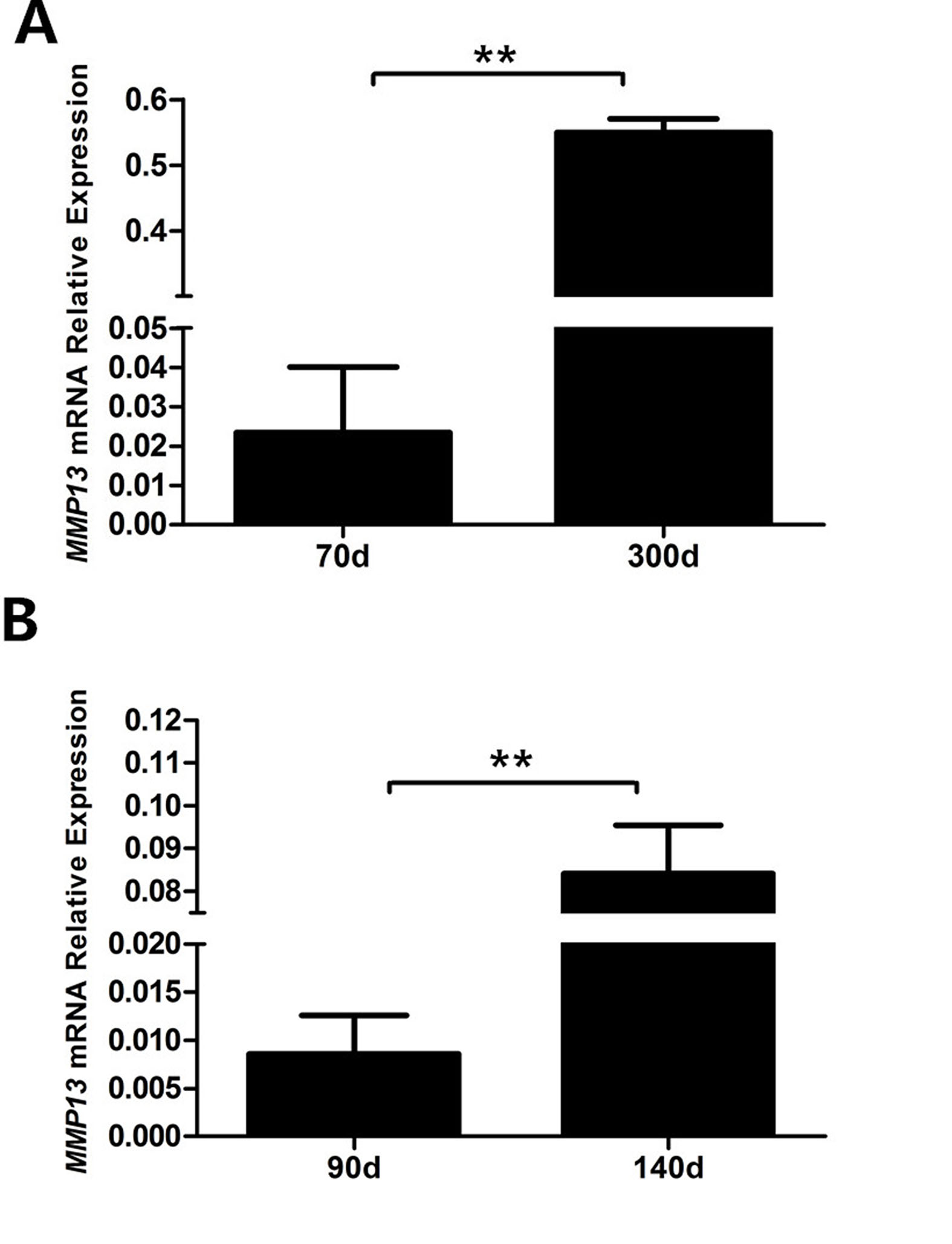

Supplement: Supplemental Material [file supp_g3.116.027755_FigureS1.jpg]

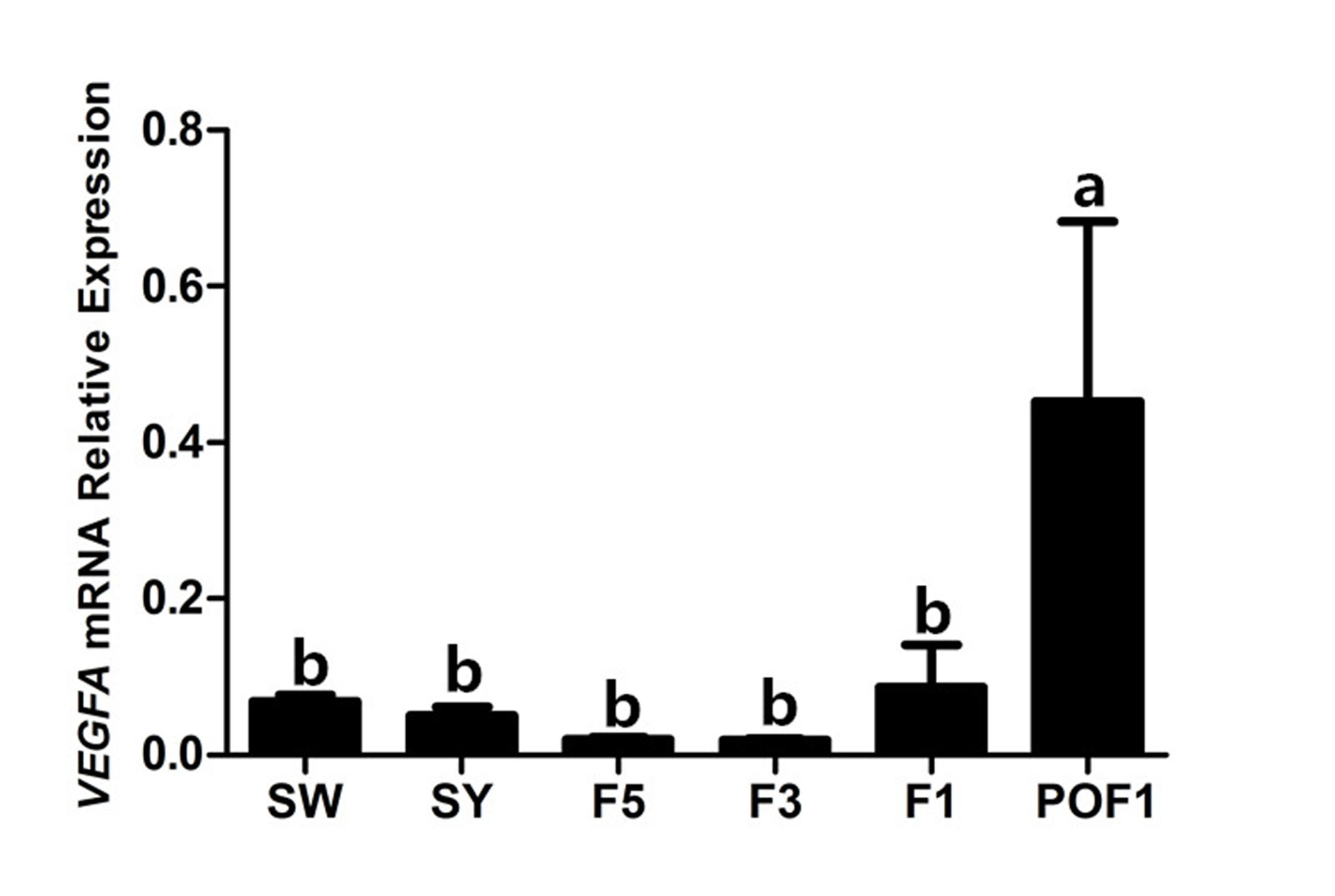

Supplement: Supplemental Material [file supp_g3.116.027755_FigureS2.jpg]
